# Impact of Orthodontic-Surgical Treatments on the Signs and Symptoms of Temporomandibular Disorders: A Systematic Review

**DOI:** 10.3390/dj12050132

**Published:** 2024-05-08

**Authors:** Elodie Ehrmann, Marie Bernabeu, Yannick Tillier, Julien Camia, Corentin Ecalle, Charles Savoldelli, Carole Charavet

**Affiliations:** 1 Université Côte d’Azur, Faculté de Chirurgie Dentaire, Département de Réhabilitation Orale, 06300 Nice, France; elodie.ehrmann@univ-cotedazur.fr (E.E.); marie.bernabeu@univ-cotedazur.fr (M.B.); 2 Centre Hospitalier Universitaire de Nice, Institut de Médecine Bucco-Dentaire, Unité Douleur et Dysfonction Orofaciales, 06300 Nice, France; 3 Biomechanics/Department of Computational Mechanics & Physics, Mines Paris, PSL University, Centre for Material Forming (CEMEF), UMR7635 CNRS, 06904 Sophia Antipolis, France; yannick.tillier@minesparis.psl.eu (Y.T.); savoldelli.c@chu-nice.fr (C.S.); 4 Université Côte d’Azur, Faculté de Chirurgie Dentaire, Département d’Orthodontie, 06300 Nice, France; julien.camia@orange.fr (J.C.);; 5 Centre Hospitalier Universitaire de Nice, Institut de Médecine Bucco-Dentaire, Unité d’Orthodontie, 06300 Nice, France; 6 Centre Hospitalier Universitaire de Nice, Institut Universitaire de la Face et du Cou, 06300 Nice, France; 7 Université Côte d’Azur, Faculté de Médecine, 06800 Nice, France; 8 Université Côte d’Azur, Laboratoire MICORALIS, UPR 7554, 06800 Nice, France

**Keywords:** orthodontic-surgical treatment, orthognathic surgery, temporomandibular disorders, joint disorders, muscle disorders

## Abstract

Introduction: Some patients exhibit temporomandibular joint or muscular disorders of the masticatory system before, during, or after orthognathic surgery (OS). These are collectively referred to as temporomandibular disorders (TMDs). This systematic literature review aimed to determine the relationship between orthodontic-surgical treatment and TMDs. Methods: An electronic search of the PubMed database, supplemented by a manual search, was performed; the search included any studies published between 2021 (date of the last search in a systematic review of the literature on the subject) and June 2023 that evaluate the prevalence of TMDs during orthodontic-surgical treatment. The diagnosis of TMDs had to be established using the diagnostic algorithm “diagnostic criteria for temporomandibular disorders (DC/TMDs)”, and the diagnosis of disc displacement had to be confirmed using magnetic resonance imaging (MRI). The data were extracted and statistically analyzed. Results: Of the 100 results, seven eligible articles were included, representing a total of 529 cases undergoing orthodontic-surgical treatment. A reduction in joint noises (64.8%), arthralgia (57 to 77%), and myalgia (73 to 100%) was found after orthodontic-surgical treatment despite the fact that a minority of patients exhibited these signs and symptoms even though they were asymptomatic before treatment. The effects of OS on disc position were objectively unpredictable. After surgery, the presence of headaches decreased without significance and the risk of their occurrence was very low (1%). The studies converged toward a reduction in the amplitudes of mouth opening and lateral/protrusion movements. Finally, after the treatment, mandibular function was improved. Conclusion: Under the conditions of this study, OS seems to have a positive impact on the signs and symptoms of TMDs; however, it is not possible to predict the consequential effects on the position of the TMJ disc, whether it is initially in a normal position or displaced.

## 1. Introduction

Orthodontic-surgical protocols have developed very rapidly in recent years in response to a sharp increase in the number of adult consultations. The orthodontist and the maxillofacial surgeon work together to define the treatment objectives, with the aim of restoring long-term skeletal, functional, and occlusal harmony in these patients. Some patients have joint or muscle disorders of the masticatory apparatus before, during or after surgery. These are known as temporomandibular disorders (TMDs) [1]. They involve the masticatory muscles, the temporomandibular joints (TMJs), and/or their associated structures. The clinical manifestations can be diverse and include noises (clicking, popping, snapping or crepitus), pain, and/or dyskinesias, most often corresponding to a limitation of the mandibular movements [2]. TMD, which affects 5 to 12% of the population, represents a significant public health issue [3]. In fact, it is the second most common musculoskeletal disorder (after lumbago) and causes pain and disability [3]. The multifactorial aetiological component of TMD includes biological (hormonal, deficiencies, systemic pathology, etc.), peripheral (anatomical, traumatic, etc.), central, behavioral, environmental, and psychosocial factors [2,4,5,6,7]. The dual Axis Diagnostic Criteria for TMD (DC/TMD) protocol provides evidence-based criteria for performing a physical diagnosis through an assessment of biobehavioral and psychosocial functioning that are an essential part of the diagnostic process [3]. The management of TMDs should be multimodal and multidisciplinary [8,9,10,11,12].

The impact of orthognathic surgery (OS) on signs and symptoms of TMDs has been extensively investigated. The bibliometric analysis by Grillo et al. [13] demonstrated a growing research interest in this field, with many publications in English and a high citation rate. The authors also stress the importance of thorough assessment, treatment, and follow-up of TMD in patients who have undergone orthognathic surgery (OS), while recognizing the need for further research and consensus on management strategies. Also, patients did not undergo the OS because they have TMDs, and so, it would be important to follow the progress of TMD in these patients. Indeed, there are several systematic reviews and meta-analyses in the literature which emphasize the lack of adequate and well-designed clinical trials. In 2009, Al-Riyami et al. [14] carried out a systematic review of the literature and concluded that although orthognathic surgery should not be recommended solely for the treatment of TMD, patients who receive orthodontic-surgical treatment and who also suffer from TMD appear to more likely see an improvement in their signs and symptoms than a deterioration. In 2013, Chauvel-Lebret et al. [15], in a systematic review on the subject, concluded that surgery had a variable and unpredictable effect on TMD. In 2017, Al-Moraissi et al. [16] conducted a systematic review and meta-analysis (including 29 studies and 5029 patients) with follow-up periods varying from 4 months to 6.3 years, and concluded that orthognathic surgery reduced TMD symptoms in many patients who had symptoms before surgery, but created symptoms in a smaller group of patients who were asymptomatic before surgery. The presence of TMD symptoms prior to surgery or the type of skeletal defect did not determine which patients had improved, unchanged, or worsened TMD after surgery. The most recent systematic review on the subject published prior to this work was that by Robin et al. [17] in 2021, which was based on the inclusion of 31 articles. The authors conclude that orthognathic surgery cannot be considered or recommended as a reliable treatment for TMD. The risk factors for joint complications are not well known; the most significant of these risk factors is probably the existence of a joint disorder prior to surgery. Rare studies have evaluated the position of the articular disc using MRI [18], while others could only infer disc displacement in the presence of the characteristic noise. Elimination of clicking after surgery may be the result of a non-reduction of the discs. Several included studies have a follow-up duration of 6 months or less; signs and symptoms might differ at longer follow-up visits.

*Ultimately*, all the studies included in these systematic reviews presented contradictory and unpredictable results: the impact of orthognathic surgery on DTM would therefore be random. As Robin et al. [17] point out, the heterogeneity of the materials and methods used (the populations studied, methods of DTM diagnosis, limited duration of patient follow-up, absence of a control group, etc.) makes it difficult to compare results and to reach conclusions.

It therefore seems important to monitor the literature on this subject. Thus, the aim of this systematic review of the literature was to assess whether any new studies investigating the link between orthodontic-surgical treatment and TMD have been published since 2021.

## 2. Methods

This systematic review was conducted closely in accordance with the PRISMA (Preferred Reporting Items for Systematic Reviews and Meta-Analyses) recommendations, which were updated in 2021 [19]. This systematic review was registered on PROSPERO (CRD42024516934).

### 2.1. Research Question and Eligibility Criteria

The aim of this systematic review was to answer the following research question: “Is orthodontic-surgical treatment a risk factor for temporomandibular disorders (TMDs)?”

Once the research question had been formulated, the definition of PICOS (Population, Intervention, Comparison, Outcome, Study design) was established:

- P (Population/Problem): Patients undergoing orthodontic-surgical treatment.
- I (Intervention): Orthognathic surgery, excluding the management of clefts or other syndromes.
- C (Comparison): Before versus after orthognathic surgery.
- O (Outcome): Signs and symptoms of TMDs.
- S (Study design): Any prospective or retrospective study, written in English and published in a peer-reviewed journal after 2021 (date of the last search in a published systematic review of the literature on the subject [15]), that evaluate the prevalence of TMDs during orthodontic-surgical treatment. TMD is diagnosed using the “diagnostic criteria for temporomandibular disorders (DC/TMDs)” diagnostic algorithm and/or, for disc displacements, magnetic resonance imaging (MRI).

The exclusion criteria included single-case reports, case series, systematic reviews and meta-analyses, narrative reviews, scoping reviews, and author/expert opinions and editorials. The various eligibility criteria are listed in Table 1.

### 2.2. Research Strategy (Electronic and Manual) and Sources of Information

To select studies that answered our research question, it was necessary to construct a search equation that would include Boolean operators and keywords; this resulted in the following equation:

(Orthognathic Surgery) AND (Temporomandibular Disorders) AND (TMD)

This equation was used in the PubMed database where the computer search was last conducted on 4 June 2023.

In addition to the electronic search, a manual search was carried out, based on all the references cited in the articles that were validated based on the title and abstract.

### 2.3. Selection of Studies

To establish the eligibility of the studies, the selected articles were assessed in three stages: the articles were selected based on their title, then their abstract, and finally, the complete reading of the article. Working independently, two reviewers took part in each stage. In the event of disagreement over the selection of an article, the two reviewers discussed their differences and reached an agreement.

### 2.4. Data Collection

The data were retrieved independently by four reviewers from the articles that met the inclusion criteria, using the PICOS approach. The following information was collected from each article: the author’s name, year and journal of publication, country, study design, patient inclusion and exclusion criteria, protocol description, primary and secondary outcome criteria, and results. It should be noted that only the data relevant to our research question were extracted.

### 2.5. Statistical Analysis of the Data

The compiled data on the prevalence of disorders, signs, or symptoms of TMD before and after surgery were compared using the Fisher test. The compiled mean values of pain intensity (masticatory muscles, temporomandibular joints, and headache; VAS on a scale of 0 to 10) or the range of mandibular movement (in mm) before and after surgery were compared using the ANOVA test.

## 3. Results

### 3.1. Literature Search

**Figure 1:**
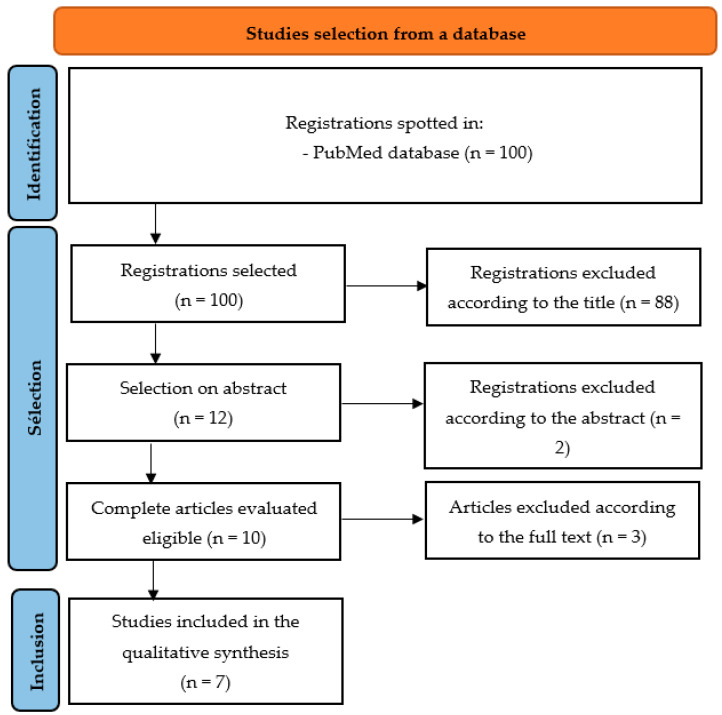
Flowchart based on PRISMA recommendations.

The search strategy detailed above yielded 100 results. Of the remaining 100, 88 articles were not selected based on their title. Of the remaining 12 articles, a new selection was made based on the abstract, and 2 articles were excluded. Ten articles were retained for a full reading of the article, and seven were included in the systematic review. The excluded articles did not meet the inclusion criteria. The details of the study selection process are shown in Figure 1 (PRISMA flow chart). None of the references cited in the articles selected according to title and abstract were retained.

### 3.2. Characteristics of the Selected Studies

#### General Characteristics

The variables relating to the characteristics of the different studies included are described in Table 2.

The characteristics of each study selected are described in Table 3 and Table 4. Table 4 lists the criteria used to assess the different studies and to address the problem.

## 4. Summary and Discussion of the Impact of Orthodontic-Surgical Treatment on TMDs

This systematic review, based on any prospective or retrospective study published after 2021 (date of the last search in a published systematic review of the literature on the subject [17]), included seven articles to answer to the following research question: “Is orthodontic-surgical treatment a risk factor for temporomandibular disorders (TMDs)?” Concerning the diagnosis of TMDs and disc displacement, all included articles were based on the diagnostic algorithm of the “Diagnostic Criteria for Temporomandibular Disorders (DC/TMDs)”, as this is the consensus standardized evaluation protocol for TMDs [3] and/or on the magnetic resonance imaging (MRI), recognized as the reference standard diagnostic method [27].

### 4.1. Population Studied

The seven studies included 570 patients, 529 of whom were undergoing orthodontic-surgical treatment (Table 2). The study by Madhan et al. [26] included a control group of 30 patients. The variables relating to the demographics of the different studies included, according to the data available, are detailed in Table 2. The study by Castro et al. [20] included a group of 19 patients off-topic, who underwent orthodontic-surgical treatments with surgical repositioning of the TMJ disc.

No study specifies the conservative treatments possibly implemented for the management of TMDs before or after OS. This constitutes a bias for the interpretation of the impact of the OS on the signs and symptoms of TMDs.

The studies were carried out in the following locations: Brazil (two studies, Bergamaschi et al. [23] and Castro et al. [20]); China (Toh et al. [21]); France (Roland-Billecart et al. [22]); India (two studies, Sahu et al. [24] and Kaur et al. [25]); and Denmark (Madhan et al. [26]). The French (Roland-Billecart et al. [22]) and Chinese (Toh et al. [21]) translations have been validated (respectively, since 2018 and 2016), but the inclusion of the French study took place prior to validation (2013 to 2015). The Danish (Madhan et al. [26]) and Indian (Sahu et al. [24] et Kaur et al. [25]) versions are currently being validated. The Portuguese (Brazilian) version has been available since 2019 but the inclusions of the two Brazilian studies (Castro et al. [20] and Bergamaschi et al. [23]) were made before this date. The version of DC/TMDs used was validated in terms of translation quality in only one study (Toh et al. [21]). This constitutes a bias in the interpretation of the results.

### 4.2. Joint Noises

Three studies [20,24,25] involving a total of one hundred and eleven patients considered the presence of preoperative joint noise and whether it persisted after surgery (Table 5). Combining these three studies, the mean percentage of patients with initial joint noise was 48.7%, whereas after surgery, the percentage was significantly reduced to 17.1% (*p* < 0.001).

The studies by Castro et al. [20] and Sahu et al. [24] also showed a significant difference in the presence of joint noise before and after OS. However, it was necessary to distinguish between the proportion of joint noises detected before the operation that persisted after the operation and the proportion that appeared after the operation. In the study by Castro et al. [20], out of the 11 patients with joint noise, no patient had noise that persisted after the operation (assessed at 16.2 months on average; between 6 and 51 months). In the study by Sahu et al. [24], out of the 28 patients with joint noise, 5 retained their clicking (at 6 months). Finally, in the study by Kaur et al. [25], out of the 15 patients with TMJ noise, 6 patients retained it after surgery (at 6 months). This means that 64.8% of the patients with a joint noise before surgery no longer had it after surgery (at 6 months), and the impact of OS on this symptom was significant according to these three studies.

Conversely, out of the eighteen patients in the study by Castro et al. [20], eight had no joint noise before surgery, and only one of these patients (12.5%) developed joint noise at more than 6 months. In the study by Sahu et al. [24], out of the 56 patients in the study, 28 had no joint noise before the operation, and 5 patients (17.85%) had a postoperative noise at 6 months. Of the 17 patients in the study by Kaur et al. [25] who did not experience a clicking sound, 2 (11.76%) did at 6 months post-op. Combining these results, out of the 57 patients with no initial clicking, 8 developed clicking after the procedure (15%; *p* = 0.55). Furthermore, the onset or resolution of joint clicking was not correlated with the patient’s initial skeletal class, nor was it correlated with the surgical technique, according to the study by Kaur et al. [25]. Elimination of clicking after surgery may be the result of non-reduction of the discs. MRI is necessary to objectively determine the position of the disc.

### 4.3. Disc Displacement

Four studies [20,21,22,26] assessed the presence of disc displacement before and after OS. Four studies [21,22,23,26] based their diagnosis of disc displacement on a set of interview and clinical examination criteria. Specificity (sp) and sensitivity (se) of these criteria (DC/TMDs) for the diagnostic of the different types of disc displacement were measured (Schiffman) (total disc displacement with reduction: se = 0.34 and sp = 0.92; total disc displacement with reduction and intermittent blocking: se = 0.38 and sp = 0.98; non-reducible disc displacement with opening limitation: se = 0.80 and sp = 0.97; and non-reducible disc displacement without opening limitation: se =0.54 and sp = 0.79). In addition to the interview and clinical examination, MRI is considered the reference standard for the diagnosis of disc displacements [3]. One of the five studies that looked at disc displacement used MRI (Table 6) [20].

Although the materials and methods of the study by Castro et al. [20] specified the absence of TMD symptoms in the preoperative phase, only 36% of the TMJs had a disc in a normal position, 41.5% had a reducible disc displacement, and 22% had an irreducible disc displacement (Table 7). This can be explained by the fact that the presence of clicking, although a sign of TMD, was not taken into account in the formation of this group; moreover, because the clicking was isolated, without other signs and symptoms, it did not justify a necessity for intervention. According to the results of this single study, which was based on MRI and a small sample size (18 patients, 36 TMJs), 38.4% of the discs in the normal pre-surgical position would shift (30.7% with reduction and 7.7% without reduction), and 26.7% of the discs shifting with reduction would reposition, while 33.33% would worsen to a shift without reduction. In other words, a reducible disc displacement before surgery was maintained in 40% of the cases after surgery. Irreducible displacements were maintained in 75% of the cases after surgery, and in the remaining 25% of the cases, the displacement became reducible. Overall, in the pre-surgery phase, 63.88% of TMJs had a displaced disc, compared with 66.66% in the post-surgery phase. This gives the impression of stability; however, in reality, of the entire group of 36 TMJs, only 20 were in the same condition after OS, and of the 23 TMJs whose discs were initially displaced, 10 improved (43.5%) and 6 worsened (26%). It is important to note at this stage that the importance of TMD symptoms (pain and functional limitation) are not always correlated with the extent of the damage (disc displacement as degenerative damage). This has led to the concept that pain in some patients with TMDs may result from altered central nervous system pain processing [5]. Studies based only on MRI will identify variable disc positions, not necessarily related to TMD symptoms [28].

The results of the studies which based their diagnosis of joint disorder on DC/TMDs are shown in Table 7. The study by Roland-Billecart et al. [22] focused on reducible disc displacements in particular and showed no significant difference before (43 out of 183 patients; 23.5%) and one year after OS (27 out of 183 patients; 14.8%). The study did, however, distinguish between the reducible displacements resolved after surgery (24 out of 43 patients: 56%; thus, 44% of the reducible displacements remained) and those that would have appeared after surgery (8 out of 138 patients: 5.8%). The only other study to have measured these proportions was that of Castro et al. [20] (Table 6), in which the reducible displacements resolved after OS represented 26.7% of the cases (which is two times less), and the disc displacements that appeared after OS represented 30.7% of the cases (which is five times more). For disc displacements, even if MRI interpretation is sometimes limited [28], the reference standard for diagnosis is MRI, and the credibility of the study by Castro et al. [20] seems superior.

In the four studies in which the diagnosis was based on the DC/TMDs [21,22,23,26], the formulated diagnostic titles were heterogeneous and often grouped a number of disorders together.

The study by Bergamaschi et al. [23] measured the number of patients with disc displacement without differentiating according to the type of displacement and revealed no significant difference before (33.33%) and after OS (26.33%).

The studies by Toh et al. [21] and Madhan et al. [26] measured the number of patients with joint disorders, including different types of disc displacement, degenerative damage, and dislocation, before OS and at 1 and 2 years after OS, respectively. The study by Toh et al. [21] followed the same group of patients before and after surgery and showed a significant reduction in these disorders (20%; *p* = 0.011), with no significant difference according to type of procedure (as detailed in Table 7), extent of surgical movement, skeletal class (I, II, or III), or presence of asymmetry. The study by Madhan et al. [26], which involved separate groups of patients, showed no significant difference in the prevalence of joint disorder before and after OS. The latter study is the only one that attempted to measure degenerative TMJ damage, and its results (with a very small sample) showed no difference before and after OS.

### 4.4. Arthralgia

Five studies [22,23,24,25,26] used the DC/TMD criteria to measure the number of patients presenting with arthralgia during orthodontic-surgical treatment, and one study [20] measured the intensity of joint pain using a visual analog scale (score from 0 to 10) (Table 8). These six studies involved 427 patients. The studies by Bergamaschi et al. [4] and Sahu et al. [24], which did not distinguish between persistent arthralgia and arthralgia that appeared after OS, nevertheless showed a significant overall reduction in the number of patients with arthralgia after surgery (*p* = 0.016 and 0.001, respectively). Three other studies [22,25,26] did not show a significant difference before and after surgery, but they all included elements pointing toward an improvement. Indeed, the study by Madhan et al. [26] tended toward an overall reduction in the prevalence of arthralgia in patients as early as 4 months after OS, and the studies by Roland et al. [22] and Kaur et al. [25] also showed, respectively, that of the 26 (14.2%) and 9 (24.3%) patients with preoperative arthralgia, 11 patients (6%) and 2 patients (5.4%) remained in the same conditions for more than 6 months, which correspond to the resolution of 57 to 77% of arthralgia after OS. Longer-term follow-up would be necessary to be able to confirm and determine the cause of the improvement in joint condition: reduction in overloads inherent to the postoperative condition, better neuromuscular coordination, good intercuspation and/or improved function. The positive psychosocial impact (axis II; paragraph 4.8) of orthodontic-surgical treatment may also have an influence on patient behavior and the progression of pain.

The study by Castro et al. [20] showed a reduction in the average intensity of joint pain using a visual analog scale in patients after the operation (at an average of 16.2 months), but without revealing any significant difference between the two measures (Table 8).

After compiling the five studies [22,23,24,25,26], which measured the prevalence of patients with arthralgia before and after OS, the mean percentage of patients with arthralgia before OS was 21.5%, whereas at more than 6 months after the intervention, this percentage was significantly reduced to 10.9% (*p* < 0.001).

Only the studies by Roland-Billecart et al. [22] and Kaur et al. [25] measured the number of patients who were free of preoperative arthralgia (185 patients in the two studies combined) and who reported arthralgia after OS (16 patients); the percentage was 8.6%.

Regarding the influence of the osteosynthesis system on the occurrence of arthralgia after OS, the study by Roland-Billecart et al. [22] did not reveal any difference linked to the rigidity of the system. The research by Kaur et al. [25] did not show any influence of skeletal class or type of procedure on the occurrence of arthralgia after OS.

### 4.5. Myalgia

Five studies [22,23,24,25,26] used the DC/TMD criteria to measure the number of patients presenting with myalgia (Table 9) during orthodontic-surgical treatment, and one study (Castro et al. [20]) measured the intensity of myalgia using a visual analog scale (score from 0 to 10). These six studies involved 427 patients (Table 9).

The study by Sahu et al. [24] showed a significant overall reduction in the number of patients with myalgia after surgery (*p* = 0.036). The other five studies [20,22,23,25,26] did not show a significant difference before and after surgery, but they all showed evidence of improvement.

The study by Castro et al. [20] showed a reduction in the average intensity of muscle pain in patients after the operation (at an average of 16.2 months), but without revealing any significant difference between the two measures (Table 9).

On the other hand, the studies by Madhan et al. [26] and Berghamaschi et al. [23], which did not discriminate between persistent myalgia and myalgia that appeared after OS, nevertheless tended toward a gradual overall decrease in the prevalence of patients with myalgia after OS. The studies by Roland et al. [22] and Kaur et al. [25], which did make this distinction, also showed that of the thirty (16%) and eight (21.6%) patients with preoperative myalgia, eight (4.4%) and zero patients, respectively, remained in the same condition for more than 6 months, which correspond to the resolution of 73 to 100% of myalgia after OS.

After compiling the five studies [22,23,24,25,26] that measured the prevalence of patients with myalgia before and after OS, the mean percentage of patients with myalgia before OS was 23.8%, whereas at more than 6 months after the procedure, this percentage was significantly reduced to 11.7% (*p* < 0.001). As for articular symptoms, longer-term follow-up would be necessary to be able to confirm and determine the cause of the improvement in muscular condition: reduction in overloads inherent to the postoperative condition, better neuromuscular coordination, good intercuspation and/or improved function.

Only the studies by Roland-Billecart et al. [22] and Kaur et al. [25] measured the number of patients who were free of preoperative myalgia (182 patients in the two studies combined) and who reported myalgia after OS (14 patients); thus, this percentage was 7.7%.

Regarding the influence of the osteosynthesis system on the occurrence of myalgia after OS, the study by Roland-Billecart et al. [22] did not show any difference linked to the rigidity of the system. The research by Kaur et al. [25] did not show any influence of skeletal class or type of procedure on the occurrence of post-OS myalgia.

### 4.6. Headaches

Four studies [22,24,25,26] measured the number of patients presenting with headaches (Table 10) during orthodontic-surgical treatment using the DC/TMD criteria (DC/TMD symptom questionnaire), and one study (Castro et al., [20]) measured the headache intensity using a visual analog scale (score from 0 to 10). These five studies involved 384 patients (Table 10).

The studies by Roland-Billecart et al. [22] and Sahu et al. [24] showed a significant overall reduction in the number of patients presenting with headaches after surgery (*p* = 0.005 and *p* < 0.001, respectively). The other three studies [20,25,26] did not show a significant difference before and after surgery, but they all showed evidence of improvement.

The study by Castro et al. [20] showed a reduction in the average intensity of the patients’ headaches after the operation (at an average of 16.2 months), but without revealing any significant difference between the two measures (Table 10).

The study by Madhan et al. [26] showed a greater reduction in the prevalence of headaches at 4 months post-OS than at 24 months.

The studies by Roland-Billecart et al. [22] and Kaur et al. [25], which differentiated between persistent headaches and headaches that appeared after OS, showed that of the eleven (6%) and eight (21.6%) patients with headaches before the operation, only one (0.55%) and zero, respectively, had them for more than 6 months, which in this small sample correspond to the resolution of 91 to 100% of the headaches. The headaches were diagnosed only using the DC/TMD symptom questionnaire; the type of headache was therefore not specified.

After compiling the four studies [22,24,25,26] which measured the prevalence of patients with headaches before and after OS, the mean percentage of patients with headaches before OS was 17.6%, whereas at more than 6 months after the intervention, this percentage had been reduced to 5.55%, with no significant difference.

Only the studies by Roland-Billecart et al. [22] and Kaur et al. [25] measured the number of patients who were free of preoperative headaches (201 patients in the two studies combined) and who reported headaches after OS (two patients); the percentage was therefore 1%.

Regarding the influence of the osteosynthesis system on the occurrence of myalgia after OS, the study by Roland-Billecart et al. [22] did not show any difference related to the rigidity of the system. The research by Kaur et al. [25] showed no influence of skeletal class or type of procedure on the occurrence of post-OS headaches.

### 4.7. Maximum Mouth Opening

Five studies measured the mean unassisted maximum mouth opening (MMO) of patients before and after surgery (Table 11). Only one study (Toh et al. [21], 64 patients) measured OBM at 4 months post-surgery and showed a significant decrease in OBM (*p* < 0.001; about 14.61 mm). There were two studies (Toh et al. [21] and Sahu et al. [24], 120 patients in total) that measured OBM at 6 months post-surgery and showed a significant decrease in OBM (from 2.27 to 7.58 mm). There were two studies (Toh et al. [21] and Bergamaschi et al. [23], 107 patients in total) that measured OBM at 1 year post-surgery and showed a significant decrease in OBM (from 4.64 to 7 mm). Conversely, the studies by Kaur et al. [25] and Castro et al. [20] showed no significant difference in OBM, respectively, at 6 months post-op and at more than 6 months up to 51 months (16.2 months on average) post-op, even though the trend was toward a decrease in amplitude (from 1.11 to 1.35 mm).

The research by Kaur et al. [25] and Toh et al. [21] did not show any influence of skeletal class and/or asymmetry or of the type of intervention on mouth opening amplitude.

### 4.8. Laterals and Propulsion

Two studies (Castro et al. [20] and Sahu et al. [24]) assessed lateral amplitudes, but only one of them [6] showed significant results in terms of amplitude reduction (*p* < 0.001) more than 6 months after the intervention (Table 12). The second study, by Sahu et al. [24], showed no significant difference in laterality but did show a significant reduction in postoperative propulsion amplitude (*p* = 0.001) at 6 months. This was the only study among the selection that measured propulsion.

### 4.9. Evolution of Mandibular Function and Psychosial Characteristics

Three studies measured the impact of orthodontic-surgical treatment on mandibular function (Table 13). The study by Castro et al. [20] assessed mandibular function using a visual analog scale, with 0 corresponding to no functional discomfort and 10 to maximum functional discomfort. Before surgery, i.e., during orthodontic treatment, the average functional discomfort (18 patients) was 1.22 ± 2.10, which decreased non-significantly to 0.22 ± 0.64 6 months after surgery.

The study by Bergamaschi et al. [23] used the OHIP-14 questionnaire to assess oral health-related quality of life. It comprised 14 questions (score out of 56) grouped into seven domains: functional limitation, physical pain, psychological discomfort, physical disability, psychological disability, social disability, and disability. The higher the score, the greater the impact on quality of life. The improvement in quality of life was significant 1 year after surgery compared with before surgery (i.e., during orthodontic treatment) (*p* < 0.001). Bergamaschi et al. [23] also studied non-specific physical symptoms including pain (NSPSIP), and non-specific physical symptoms excluding pain (NSPSEP); these symptoms were classified into three degrees (normal, moderate, and severe), then dichotomized according to the presence of suffering (moderate and severe) and the absence of suffering (normal). Compared with the pre-surgery phase, the scores improved 1 year after surgery, and they improved significantly (*p* = 0.013) for the NSPSEP. The study by Bergamaschi et al. [23] also measured depression and chronic pain before and after orthodontic-surgical treatment. The authors found that the OHIP-14 score was elevated in patients with chronic pain (*p* < 0.001), depression (*p* < 0.001), and moderate or severe NSPSIP (*p* = 0.025). Depression was associated with all domains of the OHIP-14, demonstrating its importance with regard to perceived quality of life. In brackets, some articles emphasize the importance of the patient’s psychological state before starting orthodontic-surgical treatment [29].

Madhan et al. [26] used the JFLS-8 functional limitation scale (score out of 80) which assessed mastication, mandibular mobility, and verbal and non-verbal communication in eight criteria scored from 0 (no limitation) to 10 (severe limitation). Before orthodontic treatment, the mean score was 8 ± 9. It fell to 6 ± 8 during orthodontic treatment, just before surgery, then rose to 8 ± 8 at 4 months and fell again to 3 ± 5 2 years after surgery. The improvement in function, assessed using the JFLS-8 scale, was significant between the pre-orthodontic phase and 2 years after surgery. It is probably more interesting to analyze the impact of OS on mandibular function by comparing the patient’s condition before orthodontic treatment (rather than during orthodontic treatment) with that after completion of orthodontic-surgical treatment.

To conclude this section, it is imperative to recognize the limitations inherent in this systematic literature review. First, each included study has limitations, which the authors mentioned at the end of their Discussion section. Indeed, we can cite as an example a short follow-up, a non-homogeneous distribution of patients in the different groups, statistical analysis which did not consider inter-examiner variability, etc. It should also be noted that, while most studies were prospective, two of the seven included were retrospective, and not all included a control group, which may have implications for the strength of the evidence presented.

Furthermore, the variability between studies is notable, particularly regarding the diversity of materials and methods used. Not all included studies examined each of the variables described below, and follow-up periods can be quite disparate across studies. The types of surgery performed in the studies are disparate, which makes the interpretation of the results complex. The considerable heterogeneity in sample sizes, ranging from 37 to 183 patients, with a predominance of woman participants (on average 66% woman), warrants careful consideration. Although there is some consistency in the mean age of included patients, generally between 20 and 30 years, this population is not representative of the overall population.

Given these limitations, the results of this systematic review, while offering interesting insights, should be interpreted in the context of the mentioned limitations. Addressing these limitations through further research is essential to improve knowledge in this area.

## 5. Conclusions

In conclusion, based on the present review, OS seems to have a positive impact on the signs and symptoms of TMDs, although it is not possible to predict the consequences for the position of the TMJ disc, whether initially in a normal or a displaced position. The correlation of disc position assessed by MRI with the signs and symptoms of TMDs would benefit from further studies involving more patients over a longer period.

Finally, it is essential to carry out a musculoarticular diagnosis prior to any orthodontic or surgical treatment; in the case of severe algal TMDs, it is indicated to implement appropriate management, starting by non-invasive and reversible treatment options.

## Figures and Tables

**Table 1:** Eligibility criteria.

	Inclusion Criteria	Exclusion Criteria
**Participants**	Patients following orthodontic-surgical treatment	Cleft cases or other syndromes
**Intervention**	Orthognathic surgery	Treatments for clefts or other syndromes
**Comparison**	Before versus after orthognathic surgery	
**Outcome**	Signs and symptoms of TMD. The TMD diagnosis must be made using the diagnosis algorithm “diagnostic criteria for temporomandibular disorders (DC/TMDs) “and/or magnetic resonance imaging (MRI) for disc displacements	
**Study design**	Every prospective or retrospective study, written in English, published in a journal with “peer-reviewing” after 2021 (date of the latest research from a systematic literature review published on the subject [17]), assessing the prevalence of TMDs during orthodontic-surgical treatment	Case reports, case series, systematic reviews and meta-analysis, narrative reviews, scoping reviews, author opinions/expert and editorial advice

TMDs: Temporomandibular disorders.

**Table 2:** Variables relating to the demographics of the different studies included, based on available data.

Author	Year	Country	Sample (Patients)	Age (Years)	% of Women
Castro et al. [20]	2021	Minas Gerais, Brazil	37	29.86	86%
Toh et al. [21]	2021	Hong-Kong, China	64	25.8 +/− 6.4	47%
Roland-Billecart et al. [22]	2021	Lille, France	183	Gr1: 25.9 +/− 10.9	64.50%
				Gr2: 24.6 +/− 9.4	
Bergamaschi et al. [23]	2021	Paraná, Brazil	43	18–66	76.7%
Sahu et al. [24]	2022	Chandigarh, India	56	19–35	50%
Kaur et al. [25]	2022	Chandigarh, India	37	16–35	49%
Madhan et al. [26]	2023	Aarhus, Denmark	150	Gr0: 23.8 +/− 7.8	87%
				Gr1: 22.6 +/− 4.8	
				Gr2: 24.0 +/− 5.4	
				Gr3: 24.5 +/− 4.4	
				Gr4: 25.3 +/− 5.9	

**Table 3:** General features extracted from selected studies based on the PICOS approach.

Authors	Journal/Year	Study Design	Population	Inclusion Criteria	Intervention	Comparison (Number of Patients)
Castro et al. [20]	Oral and Maxillofacial Surgery, 2021	Retrospective study	37 patients	Skeletal class II, resort to OS, with preoperative MRI and postoperative MRI more than 6 moths after surgery	OS: Mandibular advancement in anti-clockwise rotation (standard BSSO) and Lefort I +/− concomitant surgical repositioning of the disc	Comparison of 2 groups before OS and more than 6 months after surgery: Group 1 (18): OS only, without TMD symptoms, with or without disc displacement; Group 2 (19): OS + surgical repositioning of the disc, with TMD symptoms (pain or movement limitation), with disc displacement
Toh et al. [21]	Journal of Cranio-Maxillo-Facial Surgery, 2021	Prospective study	64 patients	Skeletal classes I, II, III and asymmetry, resort to OS	Mandibular OS: IVRO or SSRO or IVRO and SSRO combination +/− anterior mandibular subapical osteotomy and genioplasty; maxillary OS: Lefort I with or without segmentation	Comparison of symptoms of the same patient group (64) before OS and 1 year after (incomplete files at 3 and 6 months)
Roland-Billecart et al. [22]	Journal of Stomatology oral and maxilla-facial surgery, 2021	Retrospective study	183 patients	Skeletal classes I or II, resort to OS	OS: At a minimal Epker-type BBSO	Comparison of 2 groups regarding the osteosynthesis system/before OS and 1 year after. Gr1 (141): “rigid/hybrid”; Gr2 (42): “semi rigid” (mini titanium plates)
Bergamaschi et al. [23]	Clinical Oral Investigations, 2021	Prospective study	43 patients	Skeletal class II (ANB > 5°), resort to OS	OS: Mono- or bimaxillary sagittal Ramus osteotomy, or Lefort I +/− genioplasty	Comparison of symptoms of the same patient group (43): before OS, between 6 months and 1 year
Sahu et al. [24]	Journal of Maxillofacial and Oral Surgery, 2022	Prospective study	56 patients	Skeletal classes II or III, resort to OS	OS: Lefort I and/or BSSO	Comparison of the same patients group (56) before OS and at 6 months
Kaur et al. [25]	Journal of Cranio-Maxillo-Facial Surgery, 2022	Prospective study	37 patients	Skeletal classes II or III, resort to OS	Orthognathic surgery: Lefort I osteotomy (maxillary) and/or BSSO (mandibular advancement or retroposition in anti-clockwise rotation)	Comparison of 3 groups before OS and at 6 months: GR1 (8): cl III, maxillary advancement Lefort I; Gr2 (10): cl II BSSO of mandibular advancement +/− Lefort I; Gr3 (19): cl III, BSSO mandibular retroposition +/− Lefort I
Madhan et al. [26]	Journal of Oral Rehabilitation, 2023	Prospective study	120 patients split into 4 groups + 1 control group (30)	Skeletal classes I, II, or III, resort to OS	Bimaxillary orthodontic surgery	Comparison of the 4 groups (30 patients each): Gr 0: control, “normal” occlusion and no DFO in the past year; Gr1: OS patients before orthodontic phase initiation; Gr2: OS patients before OS; Gr3: OS patients 4 months after OS; Gr4: OS patients 24 months after OS

OS: Orthognathic surgery; IVRO: intraoral vertical ramus osteotomy; SSRO: bilateral intraoral vertical sub-sigmoid osteotomy; BBSO: bilateral sagittal split osteotomy. The exclusion criteria were as follows: craniofacial syndrome, history of orthognathic or TMJ surgery, history of facial trauma, severe systemic disease, cleft.

**Table 4:** Synthesis of judgement criteria chosen for analysis.

Authors	Purpose of the Protocol	Judgement Criteria
Castro et al. [20]	To assess the impact of orthodontic-surgical treatments, with or without surgical repositioning of the TMJ disc, on disc position and joint TMD.	Disc displacements: assessment of disc position by MRI analysis in MIO and MMO; 3 possible categories: disc in normal position, displacement with reduction, displacement without reduction; 3 experienced and blinded examiners
		Joint noises
		Headache (VAS)
		Arthralgia (VAS)
		Myalgia (VAS)
		Mandibular function (EVA)
		Maximum mouth opening (mm)
		Diductions (average right and left; mm)
Toh et al. [21]	To assess the difference in the prevalence of TMD before and after orthognathic surgery, particularly in patients with mandibular asymmetry.	TMD-related pain (myalgia, referred myofascial pain, arthralgia, headache attributed to TMDs by TMD pain screener)
		Joint disorder (disc displacement, degenerative damage, dislocation by DC/TMD examination form)
		Maximum mouth opening (mm)
Roland-Billecart et al. [22]	To evaluate the impact of the “semi-rigid” (titanium mini plates) versus “rigid/hybrid” (bi-cortical retromolar screws) osteosynthesis system on TMDs in class II or III mandibular sagittal surgery (BSSO).	Myalgia (DC/TMD)
		Arthralgia (DC/TMD)
		Reducible disc displacement (DC/TMD)
		Reducible disc displacement and intermittent locking (DC/TMD)
		Headache (DC/TMD)
Bergamaschi et al. [23]	Pre- and postoperative assessment of the impact of orthognathic surgery on oral health-related quality of life, TMDs, and psychological symptoms.	Myofascial pain (spontaneous muscle pain + 3 painful points +/− limitation < 40 mm)
		Arthralgia (RDC/TMD)
		Disc displacement (RDC/TMD)
		Maximum mouth opening (mm)
		Depression
		Chronic pain
		Non-specific physical symptoms including pain (NSPSIP)
		Non-specific physical symptoms excluding pain (NSPSEP)
		Oral health-related quality of life (OHIP14)
Sahu et al. [24]	To evaluate the effects of orthognathic surgery on TMDs.	Joint noise (DC/TMD)
		Arthralgia (DC/TMD)
		Myalgia (DC/TMD)
		Headache (DC/TMD)
		Mouth opening (mm)
		Sides (mm)
Kaur et al. [25]	To assess changes in condylar position after orthognathic surgery and their correlation with TMD symptoms.	Joint noise (DC/TMD)
		Arthralgia (DC/TMD)
		Myalgia (DC/TMD)
		Headache (DC/TMD)
		Mouth opening (mm)
Madhan et al. [26]	To measure the prevalence of TMDs during the different phases of orthodontic-surgical treatment.	Myalgia (DC/TMD)
		Arthralgia (DC/TMD)
		Degenerative joint disorder (DC/TMD)
		Joint disorder (disc displacement, degenerative damage, dislocation by DC/TMD examination form)
		Headaches
		Limitation of mandibular function (JFLS-8)

MIO: Maximum intercuspidal occlusion. MMO: Maximum mouth opening. VAS: Visual analog scale; RDC/TMD: research diagnostic criteria for temporomandibular disorder; DC/TMD: diagnostic criteria for temporomandibular disorder; JFLS-8: jaw functional limitation scale; BBSO: bilateral sagittal split osteotomy; mm: millimeters.

**Table 5:** Synthesis of study results that measured articular noises.

Authors	Patients	Skeletal Class and Type of OC (Number of Patients per Group)		Number of Patients with Joint Noise		
				Before Surgery	6 Months	*p* Value *
Castro et al. [20]	18	Cl II, standard BSSO and Lefort I		11 (61.1%)	1 (5.55%; absent preoperatively)	0.0155
Sahu et al. [24]	56	Cl II or III, Lefort I and/or BSSO		28 (50%)	10 (17.8%) corresponding to 5 (8.9%) persistent (=82% resolved) and 5 (17.85%) appearing	0.013
Kaur et al. [25]	37	Gr1 (8): cl III, Lefort I		15 (40.5%)	8 (21.62%) corresponding to 6 (16.2%) persistent (=60% resolved) and 2 (11.8%) appearing, with no significant difference between the groups	0.23
		Gr2 (10): cl II, BBSO +/− Lefort I				
		Gr3 (19): cl III, BSSO +/− Lefort I				
			Total	54/111 (48.7%)	19/111 (17.11%)	<0.001 **

* Between 6 and 51 months (16.2 months on average) for the study by Castro et al. [6]. ** *p* value obtained with a Fisher test. BBSO: bilateral sagittal split osteotomy.

**Table 6:** Description of disc position before and after OS of the 18 patients (36 TMJ) without TMD symptoms (during recruitment), from the only study in which the diagnoses are based on MRI.

Authors	Number of Patients	Skeletal Class and Type of Surgery	Position of the TMJ Disc	
			Before Surgery	6 Months to 51 Months (16.2 Months on Average)
Castro et al. [20]	18 patients (36 TMJs)	Cl II, standard BSSO and Lefort I	13 (36.1%) discs in normal position	8 (61.5.2%) in normal position
				4 (30.8%) reducible journeys
				1 (7.7%) irreducible
			15 (41.5%) reducible journeys	4 (26.7%) repositioned
				6 (40%) reducible journeys
				5 (33.3%) irreducible
			8 (22.2%) irreducible displacements	6 (75%) irreducible moves
				2 (25%) reducible
			23 (63.88%) discrete movements	24 (66.66%) discrete movements

BSSO: bilateral sagittal split osteotomy. TMJ: temporomandibular joint.

**Table 7:** Synthesis of the four studies in which the diagnosis of articular disorder is based on DC/TMDs [3].

Authors	Patients	Skeletal Class and Type of Surgery (Number of Patients)	Number of Patients with a Joint Disorder (DC/TMD)				
			Pre-Orthodontics	Pre-OS	3–4 Months	1 Year	2 Years
Toh et al. [21]	64	Cl I, II, III, and asymmetry; mandibular OS (IVRO, SSRO) +/− maxillary OS (Lefort I)	Joint disorder (disc displacement, degenerative damage, dislocation)				
				23 (35.9%)		10 (15.6%) *	
Roland-Billecart et al. [22]	183	Cl II and III; Gr1: a minima BBSO Epker, semi-rigid osteosynthesis (42); Gr2: a minima BBSO Epker, rigid osteosynthesis (141)	Reducible disc displacement				
				43 (23.5%)		27 (14.8%)	
			Reducible disc displacement and intermittent blocking				
				2 (1.1%)		8 (4.4%)	
Bergamaschi et al. [23]	43	Cl II; BSSO mono/bimaxillary or Lefort I +/− genioplasty	Disc displacement				
				14 (33.33%)		11 (26.33%)	
Madhan et al. [26]	120	Cl II and III; type of OS not described; Gr1 (30): examined before orthodontic phase; Gr2 (30): examined before OS; Gr3 (30): examined 4 months after OS; Gr4 (30): 24 months after OS	Joint disorder (disc displacement, degenerative damage, dislocation)				
			9 (30%)	11 (37%)	8 (27%)		11 (37%)
			Degenerative joint disorder				
			1 (3.5%)	1 (3.5%)	2 (7%)		3 (10%)

* Significant difference, *p* = 0.011. DC/TMD: diagnostic criteria for temporomandibular disorder; OS: orthognathic surgery; BBSO: bilateral sagittal split osteotomy IVRO: intraoral vertical ramus osteotomy; SSRO: bilateral intraoral vertical sub-sigmoid osteotomy.

**Table 8:** Result synthesis of the six studies which evaluated arthralgia.

**Authors**	**Patients**	**Skeletal Class and Type of Surgery (Number of Patients)**	**Number of Patients with Arthralgia (DC/TMD)**					***p*** Value *
			**Before OS**	**4 Months**	**6 Months**	**1 Year**	**2 Years**	
Roland-Billecart et al. [22]	183	Cl II and III; Gr1: a minima BBSO Epker, semi-rigid osteosynthesis (42); Gr2: a minima BBSO Epker, rigid osteosynthesis (141)	26 (14.2%)			11 (6%) persistent (=57% resolved) + 15 (9.6%) appeared		
Bergamaschi et al. [23]	43	Cl II; BSSO mono/bimaxillary or Lefort I +/− genioplasty	8 (18.6%)			1 (2.3%)		0.016
Sahu et al. [24]	56	Cl II or III, Lefort I and/or BSSO	27 (48.21%)		6 (10.71%)			0.001
Kaur et al. [25]	37	Cl III; Gr1 (8): cl III, Lefort I; Gr2 (10): cl II, BBSO +/− Lefort I; Gr3 (19): cl III, BSSO +/− Lefort I	9 (24.3%)		2 (5.4%) persistent (=77% resolved) + 1 (3.6%) appeared			
Madhan et al. [26]	90	Cl II and III; type of OS not described; Gr2 (30): examined before OS; Gr3 (30): examined 4 months after OS; Gr4 (30): 24 months after OS	5 (17%)	2 (7%)			2 (7%)	
		**Total**	75/349 (21.5%)		38/349 (10,9%)			<0.001
			**Intensity of arthralgia (VAS)**					
			**Before OS**		**Between 6 months and 51 months (16.2 months on average)**			
Castro et al. [20]	18	Cl II, standard BSSO and Lefort I	2.28 +/− 1.96		1.11 +/− 2.9			

DC/TMD: Diagnostic criteria for temporomandibular disorder; OS: orthognathic surgery; BBSO: bilateral sagittal split osteotomy; VAS: visual analog scale (0 to 10). * *p* values given in case of significance (<0.05).

**Table 9:** Result synthesis of the six studies which evaluated myalgia.

**Authors**	**Patients**	**Skeletal Class and Type of Surgery (Number of Patients)**	**Number of Patients with Myalgia (DC/TMD)**					***p*** Value *
			**Before OS**	**3–4 Months**	**6 Months**	**1 Year**	**24 Months**	
Roland-Billecart et al. [22]	183	Cl II and III; Gr1: a minima BBSO Epker, semi-rigid osteosynthesis (42); Gr2: a minima BBSO Epker, rigid osteosynthesis (141)	30 (16%)			8 (4.4%) persistent (=73% resolved) + 11 (6%) appeared		
Bergamaschi et al. [23]	43	Cl II; BSSO mono/bimaxillary or Lefort I +/− genioplasty	11 (25.6%)			6 (14%)		
Sahu et al. [24]	56	Cl II or III, Lefort I and/or BSSO	26 (46,4%)		11 (19%)			0.036
Kaur et al. [25]	37	Cl III; Gr1 (8): cl III, Lefort I; Gr2 (10): cl II, BBSO +/− Lefort I; Gr3 (19): cl III, BSSO +/− Lefort I	8 (21.6%)		0 persistent (=100% resolved) + 3 (8.1%) appeared			
Madhan et al. [26]	90	Cl II and III; type of OS not described; Gr2 (30): examined before OS; Gr3 (30): examined 4 months after OS; Gr4 (30): 24 months after OS	8 (27%)	6 (20%)			2 (7%)	
		**Total**	83/349 (23.78%)		41/349 (11.74%)			<0.001
			**Intensity of myalgia (VAS)**					
			**Before OS**		**Between 6 months and 51 months (16.2 months on average)**			
Castro et al. [20]	18	Cl II, standard BSSO and Lefort I	3.33 +/− 3.15		1.67 +/− 3.46			

DC/TMD: Diagnostic criteria for temporomandibular disorder; OS: orthognathic surgery; BBSO: bilateral sagittal split osteotomy; VAS: visual analog scale (0 to 10). * *p* values given in case of significance (<0.05).

**Table 10:** Result synthesis of the five studies which evaluated headaches.

**Authors**	**Patients**	**Skeletal Class and Type of Surgery (Number of Patients)**	**Number of Patients with Headache (DC/TMD)**					***p*** Value *
			**Before OS**	**4 Months**	**6 Months**	**1 Year**	**24 Months**	
Roland-Billecart et al. [22]	183	Cl II and III; Gr1: a minima BBSO Epker, semi-rigid osteosynthesis (42); Gr2: a minima BBSO Epker, rigid osteosynthesis (141)	11 (6%)			1 (0.55%) persistent		0.005
Sahu et al. [24]	56	Cl II or III, Lefort I and/or BSSO	21 (37.5%)		3 (5.35%)			<0.001
Kaur et al. [25]	37	Cl III; Gr1 (8): cl III, Lefort I; Gr2 (10): cl II, BBSO +/− Lefort I; Gr3 (19): cl III, BSSO +/− Lefort I	8 (21.6%)		0 (=100% resolved) persistent + 2 (5.4%) appeared			
Madhan et al. [26]	90	Cl II and III; type of OS not described; Gr2 (30): examined before OS; Gr3 (30): examined 4 months after OS; Gr4 (30): 24 months after OS	14 (47%)	7 (23%)			11 (37%)	
		**Total**	54/306 (17.6%)		17/306 (5.55%) **			
			**Intensity of headache (VAS)**					
			**Before OS**		**Between 6 months and 51 months (16.2 months on average)**			
Castro et al. [20]	18	Cl II, standard BSSO and Lefort I	2.94 +/− 3.22		0.67 +/− 2.05			

DC/TMD: Diagnostic criteria for temporomandibular disorder; OS: orthognathic surgery; BBSO: bilateral sagittal split osteotomy; VAS: visual analog scale (0 to 10). * *p* values given in case of significance (<0.05); ** calculated at more than 6 months.

**Table 11:** Result synthesis of the five studies which evaluated the unassisted maximum mouth opening amplitude (MMO).

**Authors**	**Patients**	**Skeletal Class and Type of Surgery (Number of Patients)**	**OBM Amplitude in mm**				***p*** Value *
			**Before OS**	**4 Months**	**6 Months**	**1 Year**	
Castro et al. [20]	18	Cl II, standard BSSO and Lefort I	44.22 ± 5.29		43.11 ± 7.21		
Toh et al. [21]	64	Cl I, II, III, and asymmetry; mandibular OS (IVRO, SSRO) +/− maxillary OS (Lefort I)	45.78 ± 7.91	31.17 ± 6.53	38.20 ± 7.84	41.14 ± 6.26	<0.001
Bergamaschi et al. [23]	43	Cl II; BSSO mono/bimaxillary or Lefort I +/− genioplasty	48			41	0.001
Sahu et al. [24]	56	Cl II or III, Lefort I and/or BSSO	46.18 ± 6.60		43.91 ± 6.28		0.004
			**Average change in OBM amplitude in mm**				
			**Before OS**	**4 months**	**6 months**	**1 year**	
Kaur et al. [25]	37	Cl III; Gr1 (8): cl III, Lefort I; Gr2 (10): cl II, BBSO +/− Lefort I; Gr3 (19): cl III, BSSO +/− Lefort I			1.35 ± 6.2		

OS: Orthognathic surgery; BBSO: bilateral sagittal split osteotomy; IVRO: intraoral vertical ramus osteotomy; SSRO: bilateral intraoral vertical sub-sigmoid osteotomy; MMO: maximum mouth opening. * *p* values given in case of significance (<0.05), obtained by comparison of pre- and post- surgery amplitudes.

**Table 12:** Result synthesis of the two studies which evaluated the amplitude of laterality and propulsion movements.

Authors	Patients	Skeletal Class and Type of Surgery	Type of Movement	Range of Movement in mm		*p* Value *
				Before OS	6 Months	
Castro et al. [20]	18	Cl II, standard BSSO and Lefort I	Lateral	8.94 ± 1.99	6.77 ± 1.74	0.001
Sahu et al. [24]	56	Cl II or III, Lefort I and/or BSSO	Lateral	6.55 ± 1.81	6.28 ± 1.6	
			Propulsion	5.99 ± 1.77	5.24 ± 1.41	0.001

OS: Orthognathic surgery; BBSO: bilateral sagittal split osteotomy; * *p* values given in case of significance (<0.05) obtained in comparison of pre- and post- surgery amplitudes.

**Table 13:** Result synthesis of the three studies which evaluated the mandibular function.

Authors	Patients	Skeletal Class and Type of Surgery	Evaluation Method	Assessment of Mandibular Function					*p* Value *
				Before Orthodontics	Before OS	4 Months	1 Year	2 Years	
Castro et al. ** [20]	18	Cl II, standard BSSO and Lefort I	EVA		1.22 ± 2.10		0.22 ± 0.64		
Madhan et al. [26]	120	Cl II and III; type of OS not described; Gr1: examined before orthodontics; Gr2 (30): examined before OS; Gr3 (30): examined 4 months after OS; Gr4 (30): 24 months after OS	JFLS	8 ± 9	6 ± 8	8 ± 8		3 ± 5	0.001
Bergamaschi et al. [23]	43	Cl II; BSSO mono/bimaxillary or Lefort I +/− genioplasty	NSPSIP		17 (40.5%)		11 (26.9%)		
			NSPSEP		19 (45.2%)		9 (22%)		0.013
			OHIP14		18		10		<0.001

OS: Orthognathic surgery; BBSO: bilateral sagittal split osteotomy; NSPSIP: non-specific physical symptoms including pain; NSPSEP: non-specific physical symptoms excluding pain; OHIP14: oral health impact profile; JFLS: jaw functional limitation scale. * *p* values given in case of significance (<0.05), obtained by comparison of the scores before orthodontics and 2 years after intervention in the study Madhan et al. [26]. ** post-surgical evaluation in the study by Castro et al. [20] happened between 6 and 51 months (16.2 months on average) after the intervention.

## Data Availability

The original contributions presented in the study are included in the article, further inquiries can be directed to the corresponding author.
